# Increased EZH2 and decreased osteoblastogenesis during local irradiation-induced bone loss in rats

**DOI:** 10.1038/srep31318

**Published:** 2016-08-08

**Authors:** Changjun Guo, Changwei Li, Kai Yang, Hui Kang, Xiaoya Xu, Xiangyang Xu, Lianfu Deng

**Affiliations:** 1 Shanghai Key Laboratory for the Prevention and Treatment of Bone and Joint Diseases with Integrated Chinese-Western Medicine, Shanghai Institute of Traumatology and Orthopedics, Rui Jin Hospital, Shanghai Jiao Tong University School of Medicine. Address: No. 197, Rui Jin Er Road, Shanghai 200025 China; 2 Department of Bone Metabolism, Institute of Radiation Medicine, Fudan University, Shanghai 200032, China. Address: No. 2094, Xietu Road, Shanghai 200032 China; 3 Department of Orthopedics, Rui Jin Hospital, Shanghai Jiao Tong University School of Medicine. Address: No. 197, Rui Jin Er Road, Shanghai 200025 China

## Abstract

Radiation therapy is commonly used to treat cancer patients but exhibits adverse effects, including insufficiency fractures and bone loss. Epigenetic regulation plays an important role in osteogenic differentiation of bone marrow mesenchymal stem cells (BMSCs). Here, we reported local bone changes after single-dose exposure to ^137^C_S_ irradiation in rats. Femur bone mineral density (BMD) and trabecular bone volume in the tibia were significantly decreased at 12 weeks after irradiation. Micro-CT results showed that tBMD, Tb.h and Tb.N were also significantly reduced at 12 weeks after irradiation exposure. ALP-positive OB.S/BS was decreased by 42.3% at 2 weeks after irradiation and was decreased by 50.8% at 12 weeks after exposure. In contrast to the decreased expression of Runx2 and BMP2, we found EZH2 expression was significantly increased at 2 weeks after single-dose ^137^C_S_ irradiation in BMSCs. Together, our results demonstrated that single-dose ^137^C_S_ irradiation induces BMD loss and the deterioration of bone microarchitecture in the rat skeleton. Furthermore, EZH2 expression increased and osteoblastogenesis decreased after irradiation. The underlying mechanisms warrant further investigation.

Patients with cancer are commonly treated using radiotherapy. Bone is among the most commonly irradiated normal tissues, and bone irradiation can lead to multiple morbidities, including fracture^1^. Insufficiency fractures are the most drastic side effects of local bone irradiation^2^. These fractures display a high rate of delayed union and nonunion, and the effect of conventional surgical treatment, including internal fixation and bone grafting, is limited^3^.

Many studies have demonstrated that the volume fraction of trabecular bone is markedly decreased in mouse radiation models^4^,^5^,^6^. Known changes in irradiated bone include osteopenia and impaired osteoblast function^7^. Decreased osteoblast activity and increased osteoclast activity result in greater bone resorption and increased trabecular bone turnover^2^. However, the specific pathogenesis of radiation-induced bone destruction remains unknown.

The differentiation of osteoblasts from bone mesenchymal stem cells (BMSCs) is an essential part of bone development. Several studies have illustrated the epigenetic mechanisms that control the transcription of key osteogenic genes and osteoblast differentiation^8^,^9^,^10^. The polycomb group protein Enhancer of Zeste Homolog 2 (EZH2) is a dependent epigenetic factor that controls osteogenesis *in vitro* and *in vivo*^11^. EZH2, which is phosphorylated by cyclin-dependent kinase 1(CDK1), plays an important role in the osteogenic differentiation of BMSCs^12^. Although the enforced expression of EZH2 in BMSCs inhibits osteogenic differentiation *in vitro* and *in vivo*^13^, no researchers have addressed the expression of EZH2 in local irradiation models. We presumed that increased EZH2 expression may cause the decreased osteoblast differentiation in BMSCs. Therefore, EZH2 represents a potential target for the treatment of bone loss induced by irradiation.

To detect the relationship between EZH2 and irradiation-induced bone loss, we established a rat bone loss model using single-dose ^137^C_S_ irradiation of the unilateral hindlimb. In addition to the bone loss and bone microarchitecture deterioration detected by micro-computed tomography at the site of exposure, we found decreased osteoblastogenesis and increased EZH2 expression in BMSCs after radiation. The underlying mechanisms warrant further investigation.

## Results

### Decreased bone mass and biomechanical quality in irradiated rat model

**Figure 1:**
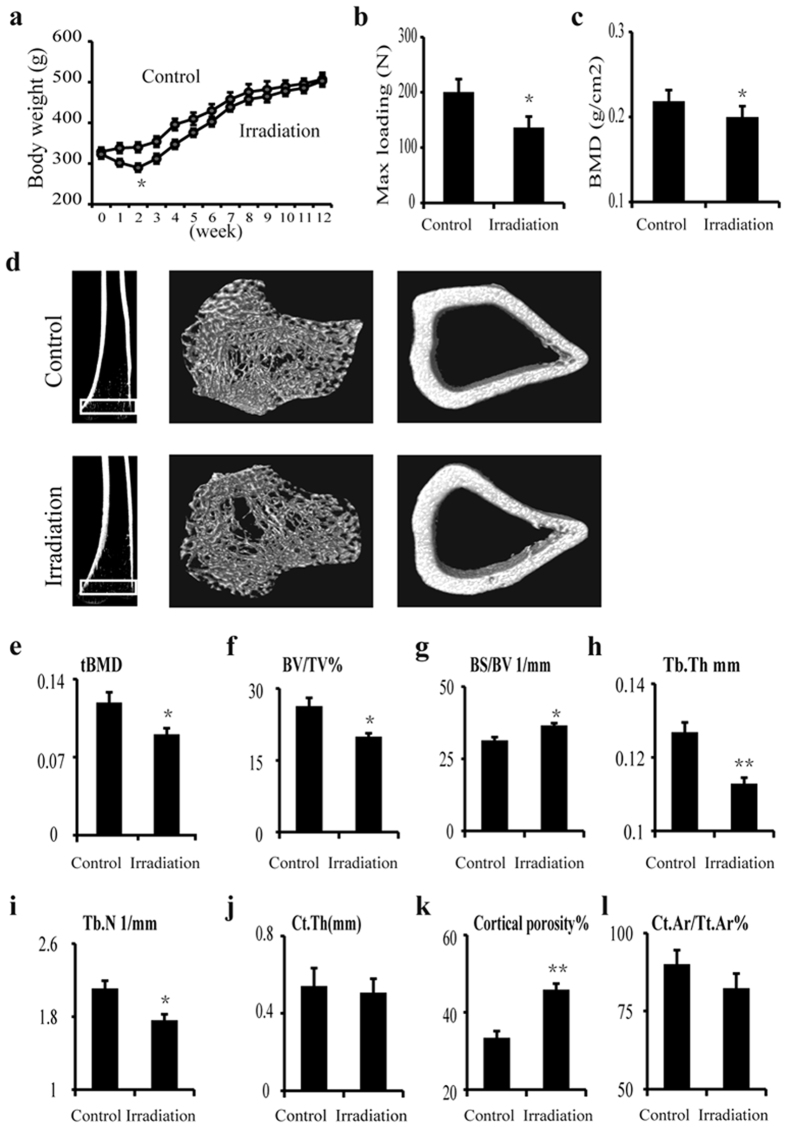
Changes in body weight and bone biomechanical quality and microarchitecture after irradiation. (**a**) Time curve of body weight changes during 12 weeks of irradiation. (**b**) Maximum loading of femurs, as analyzed using the three-point bending test at 12 weeks after irradiation. (**c**) Bone mineral density (BMD) of femurs at 12 weeks after irradiation analyzed using dual-energy X-ray absorptiometry (DXA). (**d**) Representative micro-CT images showing the microarchitectural structure of the trabecular and cortical bone regions of tibia of the control and rats at 12 weeks post-irradiation. (**e–l**) Micro-CT results showing the tBMD (**e**), BV/TV (f), BS/BV (**g**), Tb.Th (**h**), Tb.N (**i**), CtTh (**j**), cortical porosity (**k**) and Ct.Ar/Tt.Ar (**l**) values in tibia of both control and rats at 12 weeks post-irradiation. The data are presented as the mean ± standard deviation. *P < 0.05, **P < 0.01 (*n* = 16/group).

**Figure 2:**
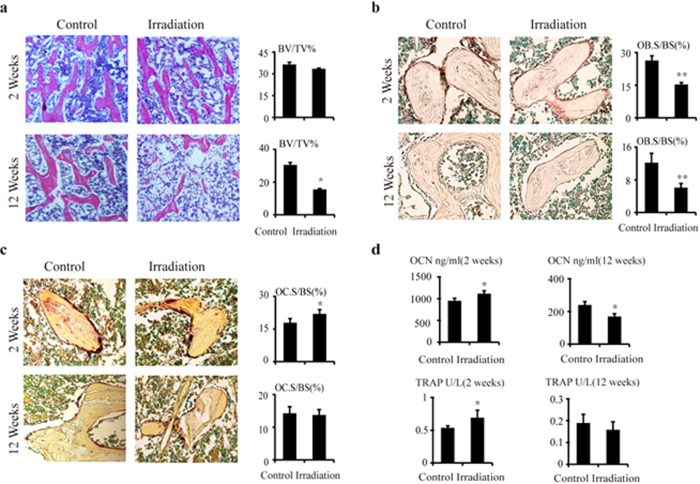
Osteoblastogenesis and osteoclastogenesis at 2 and 12 weeks after irradiation. (**a**) H&E staining of the tibia sections and bone volume fraction (BV/TV) at 2 and 12 weeks post-irradiation. Original magnification, ×200. (**b**) Alkaline phosphatase (ALP) staining of the tibia sections and osteoblast surface per bone surface (OB.S/BS,%) at 2 and 12 weeks post-irradiation. Original magnification, ×200. (**c**) Tartrate-resistant acid phosphatase (TRAP) staining and osteoclast surface per bone surface (OC.S/BS,%) at 2 and 12 weeks post-irradiation. The data are presented as the mean ± standard deviation. Original magnification, ×200. *P < 0.05 and **P < 0.01 (*n* = 8/group). (**d**) ELISA results for osteocalcin (OCN) and TRAP5b in serum at 2 and 12 weeks post-irradiation. The data are presented as the mean ± standard deviation. *P < 0.05 (*n* = 16/group).

One week after irradiation, body weight began to decline (−10.7%, *P* > 0.05) and continued to decline rapidly (−20.7%, *P* < 0.05) at 2 weeks after irradiation. However, body weight began to increase at three weeks after irradiation, and no changes were observed between the irradiated group and the control groups after 5 weeks of irradiation (Fig. 1a). The maximum loading condition of the femur was significantly reduced at 12 weeks after irradiation and was decreased by 32.6% (*P* < 0.05) in the irradiated group compared to the control group (Fig. 1b). Femur bone mineral density (BMD), as determined by dual-energy X-ray absorptiometry (DXA), was reduced at 12 weeks post-irradiation and was markedly decreased by 8.8% (*P* < 0.05) compared to the control (Fig. 1c). No differences were observed for trabecular bone volume (BV/TV%) in the tibias of irradiated rats at 2 weeks compared to controls (Fig. 2a), however, trabecular bone volume (BV/TV%) decreased significantly by 50.0% (P < 0.05) in irradiated rats at 12 weeks compared to controls (Fig. 2b).

### Changes in the bone microarchitecture after irradiation

Micro-CT was used to detect changes in bone volume and the microarchitectural structure of trabecular regions. The results showed that bone microarchitecture of the cortical tibia changed slightly, but no significant differences were observed (Fig. 1d). Twelve weeks post-irradiation (20 Gy), the trabecular BMD (tBMD) of the tibia was reduced by 24.5% (*P* < 0.05) compared to the control group (Fig. 1e), and trabecular bone volume (BV/TV) was reduced by 24.8% (*P* < 0.05) in the irradiated tibia compared to the control tibia (Fig. 1f). BS/BV was increased by 16.8% (P < 0.05) in the irradiated tibia compared to the control group (Fig. 1g). Tb.Th and Tb.N were reduced by 52.9% (*P* < 0.01) and 31.9% (*P* < 0.05), respectively, at the irradiated sites compared to the control group (Fig. 1h,i). Average cortical thickness (Fig. 1j) and cortical area fraction (Fig. 1l) showed no significant differences, however, cortical porosity increased by 95.4% (*P* < 0.01) in the irradiated tibia compared to controls (Fig. 1k).

### Decreased osteoblastogenesis after irradiation

To examine changes in osteoblast and osteoclast activity, histomorphometric analysis was performed on alkaline phosphatase (ALP)- and tartrate-resistant acid phosphatase (TRAP)-stained sections. The results showed that the ALP-positive OB.S/BS was decreased by 42.3% (*P* < 0.01) in the irradiated group compared to the control group at 2 weeks after irradiation (Fig. 2b). At 12 weeks after irradiation, a 48.4% (*P* < 0.01) reduction was found in the irradiated rats (Fig. 2b). TRAP-positive OC.S/BS was increased by 19.1% (P < 0.05) in the irradiated group compared to the controls at 2 weeks after irradiation (Fig. 2c). At 12 weeks post-irradiation, there were no significant differences between the two groups (Fig. 2c). ELISA analysis of bone turnover markers in the serum revealed a time-dependent change of bone formation marker, osteocalcin (OCN), showing a 17.3% (P < 0.05) increase at 2 weeks post-irradiation, and a 29.9% (*P* < 0.05) reduction at 12 weeks post-irradiation (Fig. 2d). The serum bone resorption marker, tartrate-resistant acid phosphatase 5b, was increased by 30% (P < 0.05) in the irradiated rats at 2 weeks post-irradiation, but was decreased by 16.7% (*P* > 0.05) in the irradiated rats at 12 weeks post-irradiation (Fig. 2d).

### Decreased expression of BMP2 and Runx2 in BMSCs after irradiation

**Figure 3:**
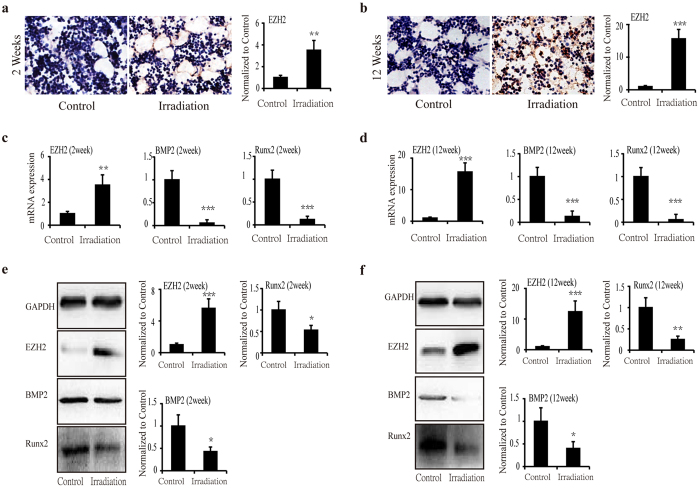
Gene and protein expression of EZH2, BMP2 and Runx2 in BMSCs at 2 and 12 weeks after irradiation. (**a**,**b**) Immunohistochemistry analysis (integrated optical density, IOD) of EZH2 expression in bone marrow at 2 weeks (**a**) and 12 weeks (**b**) after irradiation (*n* = 6/group). Original magnification, ×200. (**c**,**d**) mRNA expression of EZH2, BMP2 and Runx2 at 2 weeks (**c**) and 12 weeks (**d**) after irradiation (*n* = 6/group). (**e**,**f**) Protein expression of EZH2, Runx2 and BMP2 at 2 weeks (**e**) and 12 weeks (**f**) after irradiation (*n* = 4/group). The data are presented as the mean ± standard deviation. *P < 0.05, **P < 0.01 and ***P < 0.001.

We observed decreased osteoblastogenesis after irradiation. Therefore, we sought to evaluate the influence of irradiation on osteoblast differentiation. We determined the expression of Runx2 and BMP2 in BMSCs at 2 and 12 weeks after irradiation using real-time PCR and Western blot analyses. The results showed a significant decrease in the mRNA expression of Runx2 (88.0% in irradiated animals, *P* < 0.01) (Fig. 3c) and an immediate decline in that of BMP2 (94.5% in irradiated animals, *P* < 0.01) at 2 weeks post-irradiation (Fig. 3c). The protein expression levels exhibited similar changes to those of Runx2 and BMP2 mRNA, and were decreased by 47.0% (*P* < 0.05) and 57.0% (*P* < 0.05), respectively (Fig. 3e). At 12 weeks after irradiation, Runx2 mRNA expression decreased by 94.5% (*P* < 0.001) (Fig. 3d) in irradiated specimens, and BMP2 mRNA expression was downregulated by 87.2% (P < 0.01) in irradiated animals relative to the controls (Fig. 3d). Twelve weeks after irradiation, Runx2 protein levels and BMP2 protein levels exhibited similar changes to those of the corresponding mRNAs, and were decreased by 74.9% (*P* < 0.01) and 60.0% (*P* < 0.05), respectively (Fig. 3f).

### Increased expression of EZH2 in BMSCs after radiation

EZH2 reportedly plays an important role in the osteogenic differentiation of BMSCs^12^. We further studied EZH2 expression in tibia tissue. The immunohistochemical staining of the specimens at 2 weeks after irradiation showed positive staining of EZH2 in the nucleus, which was significantly increased (*P* < 0.01) compared to the controls (Fig. 3a). EZH2 staining was strongly positive at 12 weeks and significantly increased by 1,095% (*P* < 0.001) relative to the controls (Fig. 3b).

The increased EZH2 expression was also demonstrated by real-time PCR and western blot analyses in BMSCs. The PCR results showed that EZH2 expression was increased (248.9%, P < 0.01) at 2 weeks post-irradiation (Fig. 3c) and was sharply increased (1,136%, P < 0.001) at 12 weeks after irradiation (Fig. 3d). The Western blot results showed that relative to the control, EZH2 was increased by 364.4% (*P* < 0.001) at 2 weeks after irradiation (Fig. 3e) and by 1133.6% (*P* < 0.001) at 12 weeks after irradiation (Fig. 3f).

## Discussion

Radiotherapy-related bone complications, including osteopenia and insufficiency fractures, are commonly observed after the application of ionizing radiation in patients with cancer^14^. Many studies have illustrated the relationship between radiotherapy and bone complications^15^,^16^. In the present study, we demonstrated that localized unilateral hindlimb irradiation destroyed not only the BMD but also bone quality.

Our results demonstrated that body weight began to decline upon initial radiation, and at later time points (2 weeks), body weight was significantly different between the irradiated animals and the controls. However, at 5 weeks after irradiation, the irradiated rats had re-established a weight that was similar to that of the controls. These results are clinically relevant because the improved body weight that was detected in rats at 5 weeks after irradiation resembles the clinical post-therapy stage in humans.

The most striking side effect of radiation on bone is insufficiency fracture, which results from decreased biomechanical strength^2^. Previous studies illustrated the rapidity and severity of BMD loss in an irradiation model compared to the two common causes of osteoporosis (glucocorticoid excess-induced osteoporosis and sex steroid deficiency-induced osteoporosis)^17^,^18^,^19^. Our findings suggested that femur strength declined and radiation-induced bone loss began at 12 weeks after irradiation, a result that differs from that obtained in previous studies^17^. Many variables can account for this discrepancy, such as the source, dosage and time of the radiation used.

Previous studies showed an immediate reduction in the number of osteoblasts after radiation therapy^20^. In our study, osteoblast activity, including ALP activity, was reduced at 2 weeks after irradiation. However, OCN expression was reduced at 12 weeks after irradiation. The effects of radiation on osteoclasts are highly variable^4^,^21^,^22^,^23^,^24^. Our results showed an early increase in osteoclast activity (TRAP) at 2 weeks after irradiation, which returned to normal at 12 weeks after radiation. These results were consistent with previous studies^6^,^16^.

The mechanisms of radiation-induced decreases of osteoblastogenesis in bone remain unclear. *In vitro* studies have demonstrated that radiation suppresses the osteoblastic differentiation of BMSCs by affecting Runx2 expression^25^,^26^. BMP-2 facilitates the osteogenetic differentiation of rat BMSCs, which constitute a cell resource for tissue engineering^27^. A recent study demonstrated that EZH2 is one of the most prominent epigenetic enzymes during the osteogenic differentiation of BMSCs and mediates normal skeletal development and bone formation^11^. Runx2 up-regulation potentially suppresses EZH2 expression in mature osteoblasts^28^. EZH2 expression is connected to Runx2 activity through the LncRNA-ANCR/EZH2/Runx2 feedback loop^29^. In this study, we found that the EZH2 was highly expressed after irradiation, whereas the expression of Runx2 and BMP2 was markedly reduced. These results suggest that EZH2 may be a negative regulator of osteogenic differentiation and that EZH2 inhibition may accelerate the commitment of BMSCs to the osteogenic lineage.

Some limitations are associated with this study. First, we used single-dose radiation, which does not mimic the fractionated irradiation used clinically. Second, we used an adult rat model and mainly observed the rats at 2 and 12 weeks post-irradiation. It is unknown whether these findings are age- and/or time-dependent. Finally, further study is required to elucidate the mechanism of the increased EZH2 expression and the decreased osteoblastogenesis that are responsible for radiation-induced bone loss.

In summary, our study demonstrates that localized radiation directly affected bone loss, including the loss of BMD and bone strength. In our focal radiation rat model, the suppressed osteoblast differentiation of BMSCs may be the major cause of this bone loss. EZH2, a key regulator of osteogenic differentiation, may be responsible for the mechanisms that control osteogenesis in radiation-induced bone loss. These findings might present a novel target for epigenetic drugs that can be used to treat the bone loss associated with localized radiotherapy.

## Materials and Methods

### Ethics statement

All animal experiments were performed according to the protocol approved by the Shanghai Jiao Tong University Animal Care and Use Committee and in direct accordance with the animal care guidelines of the Ministry of Science and Technology of the People’s Republic of China. The protocol was approved by Shanghai Jiao Tong University Animal Care and Use Committee. All surgeries were performed under anesthesia, and all efforts were made to minimize suffering.

### Animal treatment

Four-month-old male Sprague-Dawley rats (Shanghai Lab Animal Resource Center, STCSM, Shanghai, China) were used in the experiments. Rats in the irradiated group (n = 20) were subjected to ketamine anesthesia. The rats were placed into a ^137^Cs γ-ray irradiation chamber and exposed to 20 Gy radiation on the right side of the proximal tibia and distal femur (0.8 Gy/minute for 25 minutes, ^137^Cs γ-ray irradiation machine). Control rats (n = 20) were similarly manipulated and anesthetized before undergoing sham irradiation (0 Gy). All experiments involving animals were performed according to institutionally approved and current animal care guidelines. Animals were euthanized at 2 or 12 weeks after irradiation, and tissues including tibia, femur and blood were collected for analysis.

### Bone mineral density (BMD) analysis

Densitometry was performed by dual-energy X-ray absorptiometery (DXA). High-resolution scans of the femur were obtained at 12 weeks after irradiation. In brief, the isolated femurs were placed in the same location on the platform of a dual-energy X-ray absorptiometer (Discovery A, Hologic Inc., Bedford, MA, USA) and scanned using high-resolution imaging as adapted for the BMD measurement of small-animal skeletal subregions according to the manufacturer’s instructions.

### Evaluation of bone biomechanical quality

Bone biomechanical quality was evaluated using the three-point bending test at 12 weeks after irradiation (femur). The tests were performed on an electronic universal material testing machine (INSTRON-5543, USA) linked to Merlin software according to the manufacturer’s instructions. The maximum load of the femur was obtained automatically from the load-strain curve in the three-point bending test using a span of 18 mm and a loading speed of 10.0 mm/min.

### Micro-CT analysis

Micro-CT analysis of the tibia (at 12 weeks post-irradiation) was performed using a SkyScan-1176 micro-computed tomography (μCT, Bruker Micro-CT, Belgium) system. Scans were performed using a PANalytical Microfocus Tube (17.93 μm voxel size, 65 kV, 385 μA and 0.5-degree rotation step. 180-degree angular range). Micro-CT evaluation of the trabecular bone was performed on a 2-mm region of the metaphyseal spongiosa in the proximal tibia. The regions were located 0.5 mm above the growth plate. Cortical bone measurements were performed on a 1-mm region of the mid-diaphysis of the tibia. NR Econ software version 1.6 was used for the 3D reconstruction and viewing of images. After 3D reconstruction, the CT software version 1.13 was used for bone analysis. The specific abbreviations of the indices defined in the results are noted in Table 1.

### Histological examination and histomorphometry

The tibias (at 2 and 12 weeks post-irradiation) were removed and fixed in PLP fixative (2% paraformaldehyde containing 0.075M lysine and 0.01 M sodium periodate) for 3 days at 4 °C and processed histologically as previously described^30^. The distal 1/3 of the tibias were decalcified in ethylenediaminetetraacetic acid (EDTA) glycerol solution for 28–30 days at 4 °C. The decalcified bones were dehydrated and embedded in paraffin, after which 5-μm sections were cut using a rotary microtome. The sections were stained with hematoxylin and eosin (H&E) and histochemically analyzed for alkaline phosphatase (ALP) activity using a BCIP/NBT kit (Beyotime Biotechnology, China) and for tartrate-resistant acid phosphatase (TRAP) activity using a TRACP kit (Sigma, USA). The sections were then counterstained with methyl green and mounted in Kaiser’s glycerol jelly. The following parameters were measured: trabecular bone volume per tissue volume (BV/TV, %) for bone mass, the osteoblast surface per bone surface (OB.S/BS, %) for bone formation, and the osteoclast surface per bone surface (OC.S/BS, %) for bone resorption.

### ELISA measurement of bone turnover markers in serum

Blood was collected at 2 and 12 weeks post-irradiation, and serum from each rat was analyzed individually in duplicate for the bone formation marker osteocalcin (OCN) using the Rat Osteocalcin ELISA Kit (Immunodiagnostic Systems Inc., England). Bone resorption was analyzed based on the marker for tartrate-resistant acid phosphatase 5b (TRAP5b) using the Rat TRAP Assay (Immunodiagnostic Systems Inc.,England) following the instructions included with the assay. The average value of the duplicate measurements was obtained for each rat.

### Immunohistochemistry and evaluation

Immunohistochemistry was performed as previously described^31^. Decalcified gelatinized tibia sections were boiled in 10 mM sodium citrate (pH 6.0) for 5 min to retrieve the antigen. The sections were then quenched with 3% hydrogen peroxide for 15 min to reduce endogenous peroxide activity and blocked with 2% BSA in PBS. The sections were then incubated with rabbit anti-EZH2 primary antibody or rabbit IgG as controls (1:1,00 dilution,Cell Signaling Technology, Danvers, MA). Nuclei were counterstained with hemalum (FARCO Chemical Supplies, Hong Kong). The slides were visualized under a microscope (ZEISS, AXIO).

Immunohistochemistry analysis (integrated optical density, IOD) was quantitated using IPP 6.0 image analysis software (Media Cybernetics, USA), 5–8 fields of view were selected on each section and photographed. Image analyses were performed as previously described^32^. The irradiation group was normalized to the controls, and fold changes are shown in the histogram.

### Cell culture

BMSCs of the tibia and femur (at 2 and 12 weeks post-irradiation) were flushed out with a-MEM (Gibco BRL, Carlsbad, CA). Cells were seeded on 100-mm culture dishes (Nunc, Rochester, NY), and cultured in a-MEM supplemented with 100 IU/mL penicillin, 100 mg/mL streptomycin (Gibco BRL), and 10% fetal bovine serum (FBS, Gibco BRL). Medium was replaced every 3–4 days to remove non-adherent hematopoietic cells. At 2 weeks after irradiation, the adherent cells were collected and sorted by flow cytometry as CD29, CD90 and CD34. The sorted BMSCs (CD29^+^, CD90^+^, CD34^−^) were cultured in fresh medium and further subcultured. The first passage of sorted BMSCs was termed passage 1. BMSCs from passages 3 and 5 were used in the experiments.

### Quantitative real-time PCR

BMSCs from passage 3 were collected, and the total RNA was extracted using TRIzol reagent (15596, Invitrogen, Carlsbad, CA) according to the manufacturer’s protocol. Total RNA was reverse transcribed to cDNA using the QuantiTect Rev Transcription Kit (205311, Qiagen, Chatsworth, CA). The number of cDNA molecules in the reverse-transcribed samples was determined by real-time PCR analyses using a modified method and the QuantiTect SYBR Green PCR Kit (204143, Qiagen) on the Mx3000P Real-Time PCR system (Stratagene, La Jolla, CA). The primers were obtained from SBS Gene (www.sbsgene.com) using a previously described protocol. The following sequences were retrieved: EZH2, 5′-GGGAGAGAACAACGATAAAGAAGAAGA-3′ and 5′-GGCTTCATCTTTATTGGTGTTTGACA-3′ (99 bp, XM_006236408.2), bone morphogenetic protein 2 (BMP2), 5′-TAGTGACTTTTGGCCACGACG-3′ and 5′- GCTTCCGCTGTTTGTGTTTG-3′ (81 bp, XM_008762246.1), Runt-related transcription factor 2 (Runx2), 5′-AGCCTCTTCAGCGCAGTGAC-3′ and 5′-CTGGTGCTCGGATCCCAA-3′ (132 bp, AF187319), GAPDH,5′-AAACCCATCACCATCTTCCA-3′ and 5′-GTGGTTCACACCCATCACAA-3′ (198 bp, DQ403053). The reaction volume comprised 12.5 μL of master SYBR Green I, 0.25 μM of each 5′ and 3′ primer, and 2 μL of samples or H_2_O. H_2_O was added to a final volume of 25 μL. A melting curve was obtained at the end of each run to discriminate specific from nonspecific cDNA products. The cDNA content was normalized by subtracting the cycle numbers of GAPDH from those for the target gene (∆Ct = Ct of target gene − Ct of GAPDH), and the gene expression level was calculated using 2^−(∆Ct)^.

### Western blotting analysis

BMSCs from passage 4 were collected and lysed in RIPA buffer (P0013B, Beyotime, Jiangsu, China). The cells were extracted for 20 min on ice. Insoluble materials were removed by centrifuging at 12,000 x *g* for 30 min. The supernatants were collected, and the amount of protein in the supernatants was quantified using the BCA protein assay (P0012, Beyotime) using BSA as a standard. The sample protein was denatured in SDS-PAGE sample loading buffer (P0015, Beyotime) in boiling water for 5 min. Aliquots of the samples (40 μg) were then resolved using SDS-PAGE in 12% gels under reducing conditions and electroblotted onto PVDF membranes (Ipvh00010, Millipore, Bedford, MA). The membranes were blocked with 5% fat-free dry milk in TBST (0.1% Tween-20 and 0.1 M NaCl in 0.1 M Tris–HCl, pH 7.5) for 2 h at room temperature and with rabbit anti-EZH2 antibody (5246s, Cell Signaling Technology, USA, 1:1,000), rabbit anti-BMP2 antibody (13105s, Cell Signaling Technology, USA, 1:1,000), goat anti-Runx2 antibody (ab56326, Abcam, Cambridge, MA, 1:1,000) and mouse anti-GAPDH antibody (Kangcheng, Shanghai, China, 1:5,000) at 4 °C overnight. The membranes were then incubated with horseradish peroxidase-conjugated secondary antibody (1:5,000, Santa Cruz Biotechnology, Santa Cruz, CA) at room temperature for 1 h and then analyzed using chemiluminescence detection (P0018, Beyotime). Each incubation step was followed by three washes (10 min each) with TBST. The protein bands were quantitatively analyzed using an image analysis system (QuantityOne software, Bio-Rad, Hercules, CA, USA).

### Statistical analysis

All data are presented as the mean ± standard deviation. We used two-tailed *t*-tests to determine the significance between two groups. We analyzed the results of multiple groups by one-way or two-way ANOVA with Bonferroni post-hoc tests using SPSS version 17 software. For all statistical tests, we considered a *P* value < 0.05 statistically significant.

## Additional Information

**How to cite this article**: Guo, C. *et al*. Increased EZH2 and decreased osteoblastogenesis during local irradiation-induced bone loss in rats. *Sci. Rep.* **6**, 31318; doi: 10.1038/srep31318 (2016).

## Figures and Tables

**Table 1:** Definitions and Descriptions of 3D Outcomes of the Trabecular Bone Microarchitecture.

Abbreviation	Variable description
tBMD	Trabecular bone mineral density
BS/BV	Ratio of the segmented bone surface to the segmented bone volume
BV/TV	Ratio of the segmented bone volume to the total volume of the region of interest
Tb/Th	Mean thickness of trabeculae, assessed using direct 3D methods
TbSp	Mean distance between trabeculae, assessed using direct 3D methods
TbN	Average number of trabeculae per unit length
CtTh	Average cortical thickness
Ct.Ar/Tt.Ar	Cortical area fraction
